# Supplementary material for: Trust Is for the Strong: How Health Status May Influence Generalized and Personalized Trust
Source: Healthcare (Basel). 2023 Aug 23;11(17):2373. doi: 10.3390/healthcare11172373 (PMC10486567; doi:10.3390/healthcare11172373)
Supplement: Supplementary file 1 [file healthcare-11-02373-s001.zip › healthcare-2471484-supplementary.pdf]

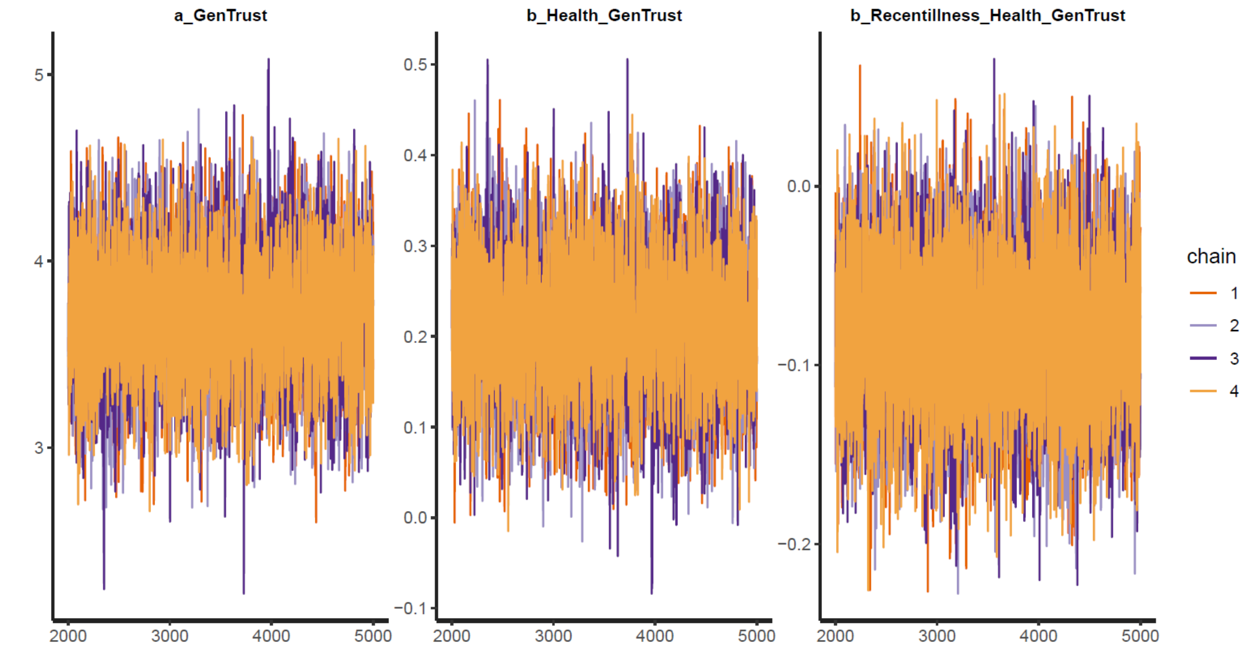

Figure S1. Model 1's trace plots

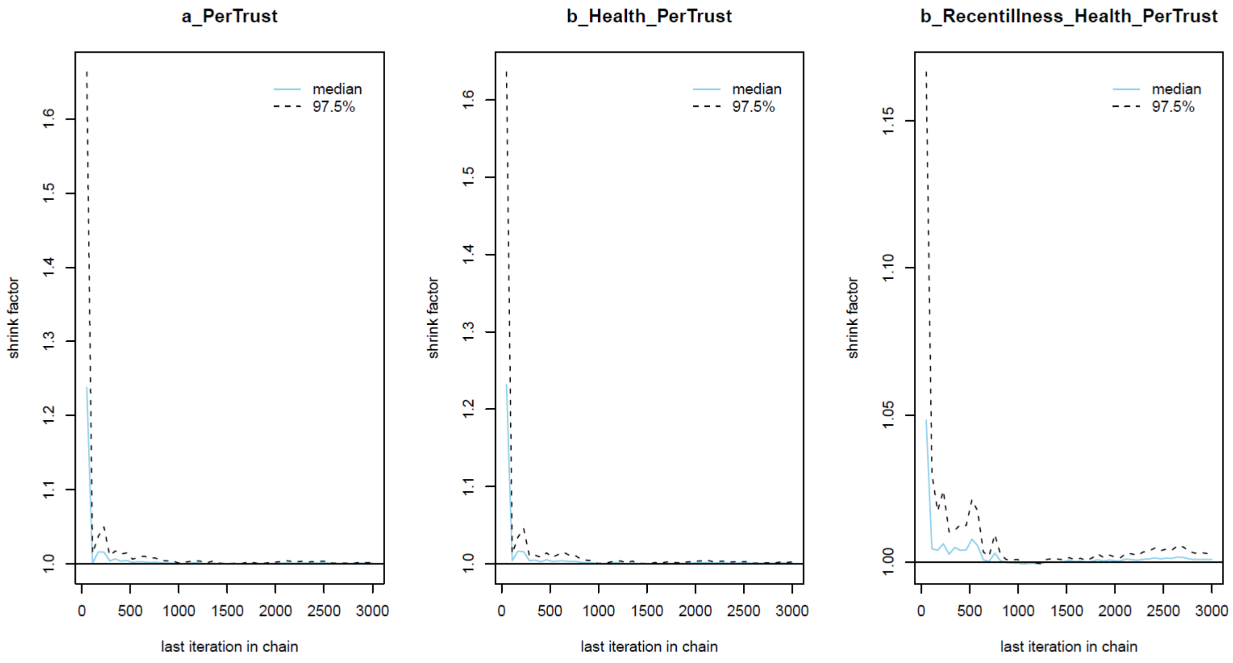

Figure S2. Model 1's Gelman-Rubin-Brooks plots

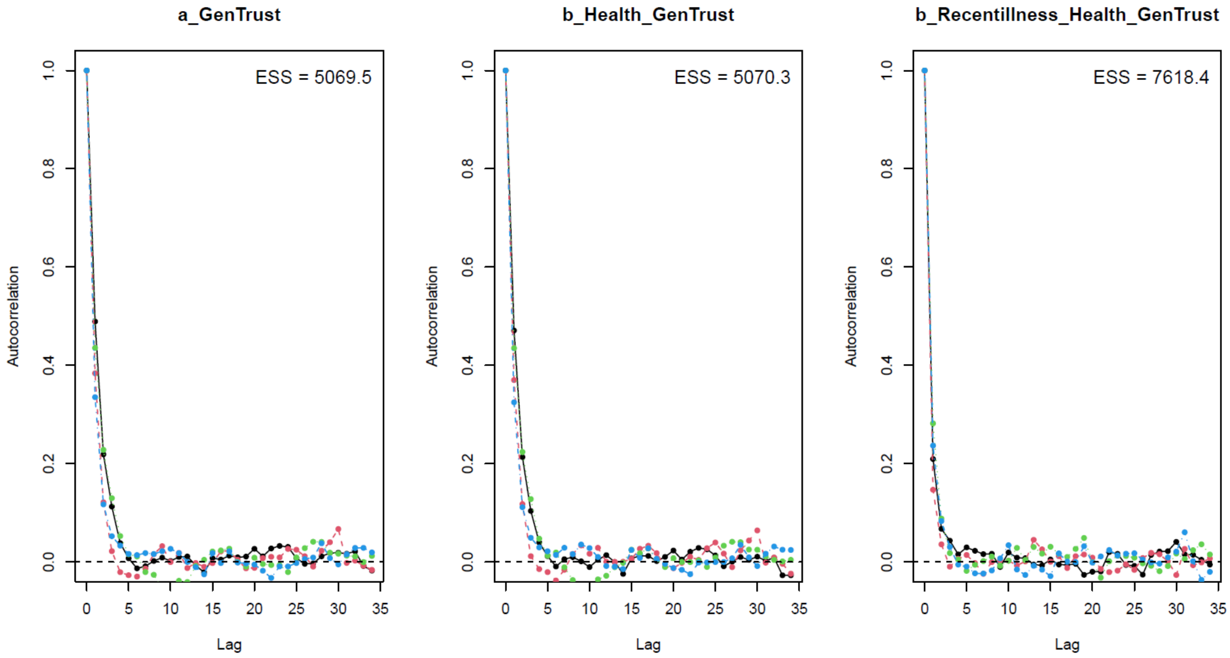

Figure S3. Model 1's autocorrelation plots

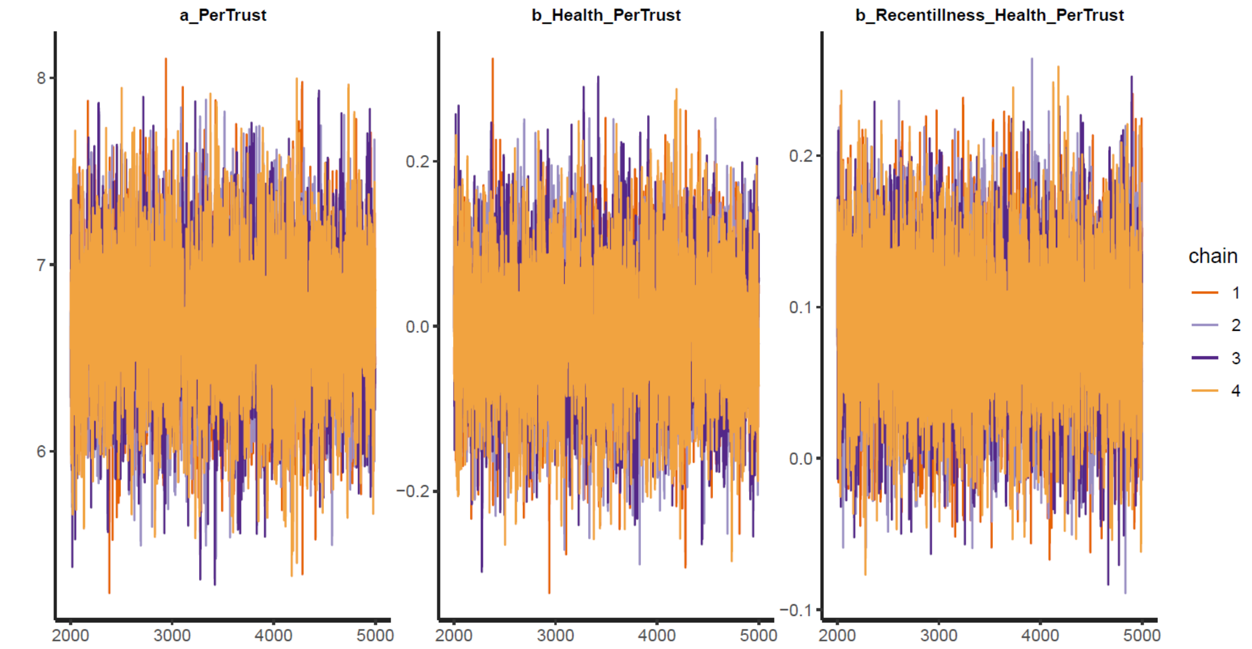

Figure S4. Model 2's trace plots

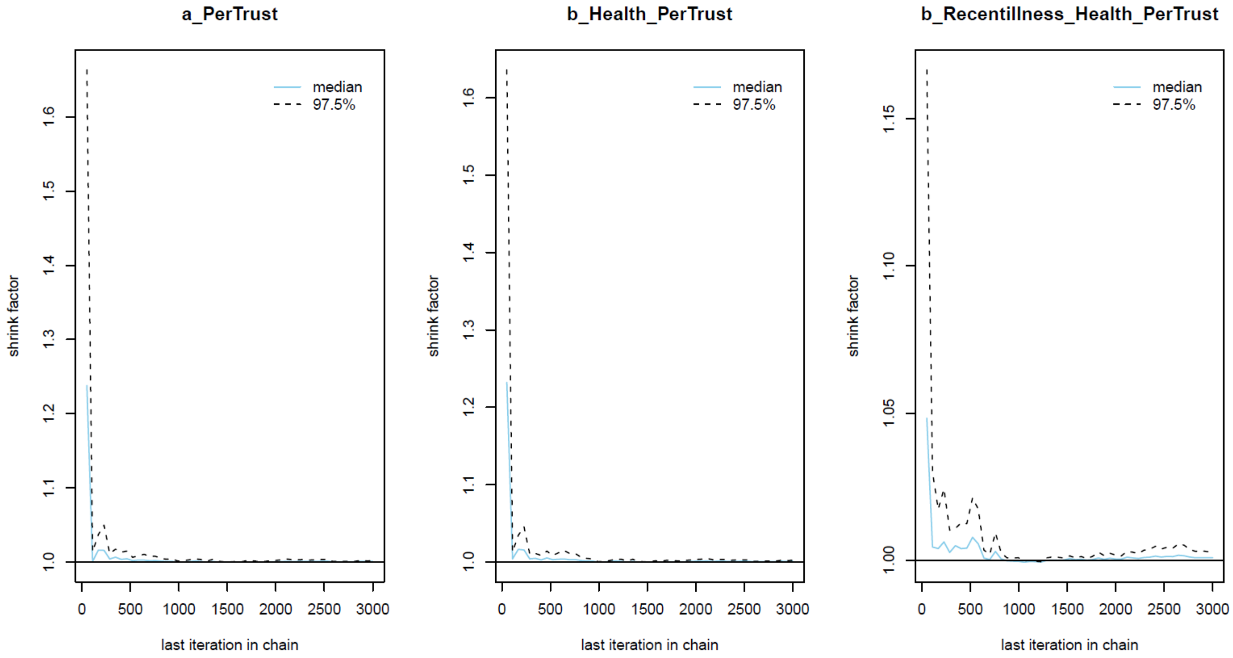

Figure S5. Model 2's Gelman-Rubin-Brooks plots

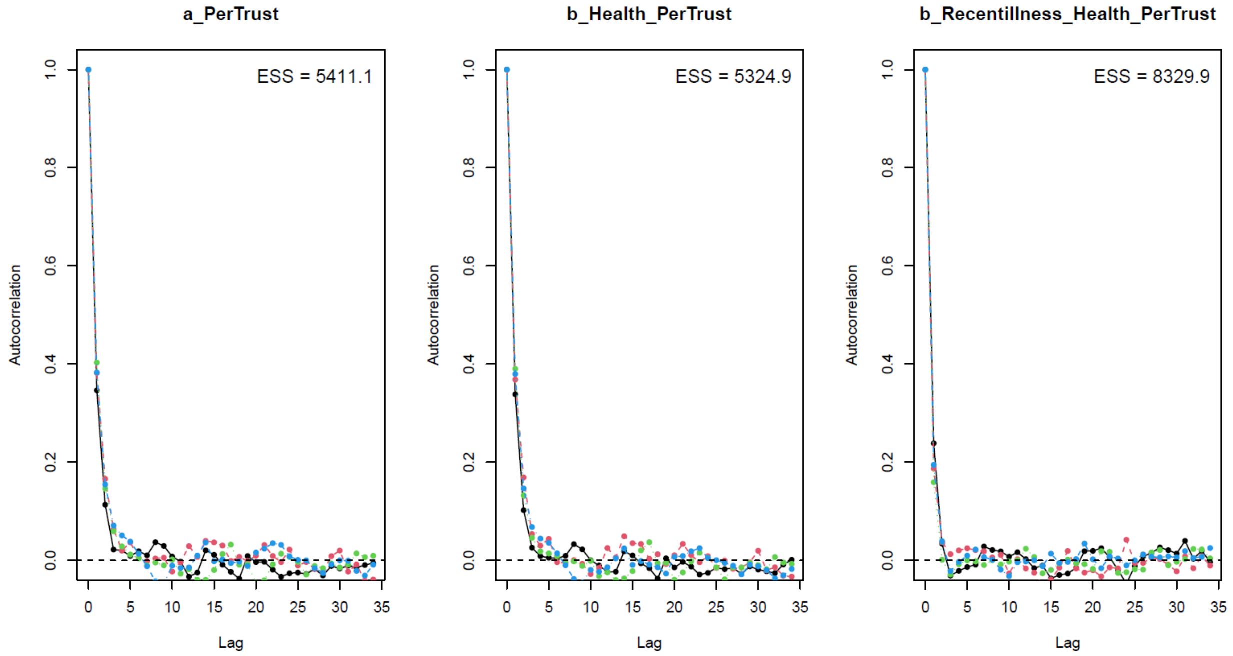

Figure S6. Model 2's autocorrelation plots
